# PDL1 Regulation by p53 via miR-34

**DOI:** 10.1093/jnci/djv303

**Published:** 2015-11-17

**Authors:** Maria Angelica Cortez, Cristina Ivan, David Valdecanas, Xiaohong Wang, Heidi J. Peltier, Yuping Ye, Luiz Araujo, David P. Carbone, Konstantin Shilo, Dipak K Giri, Kevin Kelnar, Desiree Martin, Ritsuko Komaki, Daniel R. Gomez, Sunil Krishnan, George A. Calin, Andreas G. Bader, James W. Welsh

**Affiliations:** **Affiliations of authors:** Departments of Experimental Radiation Oncology (MAC, DV, XW, YY), Experimental Therapeutics (CI, GAC), and Radiation Oncology (RK, DRG, SK, JWW), The University of Texas MD Anderson Cancer Center, Houston, TX; Mirna Therapeutics, Inc., Austin, TX (HJP, KK, DM, AGB); Ohio State University, Columbus, OH (LA, DPC, KS); Texas Veterinary Pathology Associates (Houston), Houston, TX (DKG).

## Abstract

**Background:**

Although clinical studies have shown promise for targeting PD1/PDL1 signaling in non–small cell lung cancer (NSCLC), the regulation of PDL1 expression is poorly understood. Here, we show that PDL1 is regulated by p53 via miR-34.

**Methods:**

p53 wild-type and p53-deficient cell lines (p53^–/–^ and p53^+/+^ HCT116, p53-inducible H1299, and p53-knockdown H460) were used to determine if p53 regulates PDL1 via miR-34. PDL1 and miR-34a expression were analyzed in samples from patients with NSCLC and mutated p53 vs wild-type p53 tumors from The Cancer Genome Atlas for Lung Adenocarcinoma (TCGA LUAD). We confirmed that PDL1 is a direct target of miR-34 with western blotting and luciferase assays and used a p53^R172HΔ^g/+K-ras^LA1/+^ syngeneic mouse model (n = 12) to deliver miR-34a–loaded liposomes (MRX34) plus radiotherapy (XRT) and assessed PDL1 expression and tumor-infiltrating lymphocytes (TILs). A two-sided *t* test was applied to compare the mean between different treatments.

**Results:**

We found that p53 regulates PDL1 via miR-34, which directly binds to the *PDL1* 3’ untranslated region in models of NSCLC (fold-change luciferase activity to control group, mean for miR-34a = 0.50, SD = 0.2, *P* < .001; mean for miR-34b = 0.52, SD = 0.2, *P* = .006; and mean for miR-34c = 0.59, SD = 0.14, and *P* = .006). Therapeutic delivery of MRX34, currently the subject of a phase I clinical trial, promoted TILs (mean of CD8 expression percentage of control group = 22.5%, SD = 1.9%; mean of CD8 expression percentage of MRX34 = 30.1%, SD = 3.7%, *P* = .016, n = 4) and reduced CD8^+^PD1^+^ cells in vivo (mean of CD8/PD1 expression percentage of control group = 40.2%, SD = 6.2%; mean of CD8/PD1 expression percentage of MRX34 = 20.3%, SD = 5.1%, *P* = .001, n = 4). Further, MRX34 plus XRT increased CD8^+^ cell numbers more than either therapy alone (mean of CD8 expression percentage of MRX34 plus XRT to control group = 44.2%, SD = 8.7%, *P* = .004, n = 4). Finally, miR-34a delivery reduced the numbers of radiation-induced macrophages (mean of F4-80 expression percentage of control group = 52.4%, SD = 1.7%; mean of F4-80 expression percentage of MRX34 = 40.1%, SD = 3.5%, *P* = .008, n = 4) and T-regulatory cells.

**Conclusions:**

We identified a novel mechanism by which tumor immune evasion is regulated by p53/miR-34/PDL1 axis. Our results suggest that delivery of miRNAs with standard therapies, such as XRT, may represent a novel therapeutic approach for lung cancer.

TP53, also known as p53, is one of the most commonly mutated genes in cancer (1). It is critical in regulating cell division, apoptosis, senescence, and DNA damage and repair (2–4). p53 is also important for modulating the immune response (5–9). However, whether p53 is involved in tumor immune evasion is poorly understood. This topic is particularly relevant for several reasons, among them evidence linking microRNAs (miRNAs), p53, and adaptive and innate immunity (6,10). For instance, several p53-regulated miRNAs have been implicated in adaptive and innate immunity, including the miR-17~92 cluster (11), miR-145 (12), and let-7 (13). Importantly, p53 can regulate tumor cell recognition by natural killer (NK) cells via the p53-regulated miRNA miR-34a (10). We recently showed that the miR-200 family, another miRNA family regulated by p53 (14), directly regulates PDL1 (programmed death 1 ligand 1; also known as B7-H1 or CD274) (15). PDL1 is overexpressed in many human cancers, including non–small cell lung cancer (NSCLC) (16–18), promoting T-cell tolerance and escape host immunity (19). Early clinical trials using monoclonal antibodies that block the PD1/PDL1 interaction have shown promise in some patients with advanced cancer (20,21).

Here, we investigated the potential role of p53 in regulating PDL1 expression in NSCLC. We found that p53 regulates PDL1 via miR-34 by using a series of experiments involving lung cancer cell lines, miRNA target-predicting databases, and tissue samples from patients with NSCLC. Using a syngeneic mouse model of lung cancer, we demonstrated that MRX34, a liposomal formulation complexed with miR-34a mimics that is currently the subject of a phase I clinical cancer trial (22–24), alone or in combination with radiotherapy (XRT), reduced PDL1 expression in the tumor and antagonized T-cell exhaustion.

## Methods

A complete description of the methods, including cell lines, establishment of stable p53–knockdown cells, transfection, quantitative polymerase chain reaction, luciferase assay, chromogenic in situ hybridization for miR-34a, immunohistochemical analysis of PDL1, isolation of tumor-infiltrating T-cells, and assays for TNFα and IFNγ, is presented in the Supplementary Materials (available online).

### Syngeneic Subcutaneous Model

All mouse studies were approved by the Institutional Animal Care and Use Committee (IACUC) of The University of Texas MD Anderson Cancer Center before their initiation; animal care was provided according to IACUC standards, and all mice had been bred and were maintained in our own specific pathogen-free mouse colony. To create the tumors, syngeneic male 129/Sv mice three to four months of age were injected subcutaneously in the right flank with 10^6^ 344SQ murine lung adenocarcinoma cells (a lung cancer cell line derived from a spontaneous subcutaneous metastatic lesion in p53R172HΔg/+K-rasLA1/+ mice) (25) (12 mice per group). Detailed information regarding treatment regimen is presented in the Supplementary Materials (available online).

### NSCLC Patient Samples

The study was approved by The Ohio State University Cancer Institutional Review Board (protocol # 2013C0014), and informed consent was obtained from each patient. Samples were analyzed using chromogenic in situ hybridization for miR-34a analysis and immunohistochemical analysis of PDL1 expression.

### Statistical Analysis

Data analyses were carried out by using Graph Pad (GraphPad Prism, La Jolla, CA), Excel (Microsoft Corp, Redmond, WA), or R (version 3.0.1, http:///www.r-project.org/), and *P* values of less than .05 were considered statistically significant. All statistical tests were two-sided. Data from The Cancer Genome Atlas for Lung Adenocarcinoma (TCGA LUAD) were downloaded from TCGA portal (http://tcga-data.nci.nih.gov/). Somatic mutations in *p53* were retrieved from cbioPortal (http://www.cbioportal.org/). The Spearman’s rank-order correlation test was applied to measure the strength of the association between p53 and PDL1 (CD274) mRNA levels. Further details on data analyses are provided in the Supplementary Methods (available online).

## Results

### P53 Regulation of PDL1

**Figure 1.**
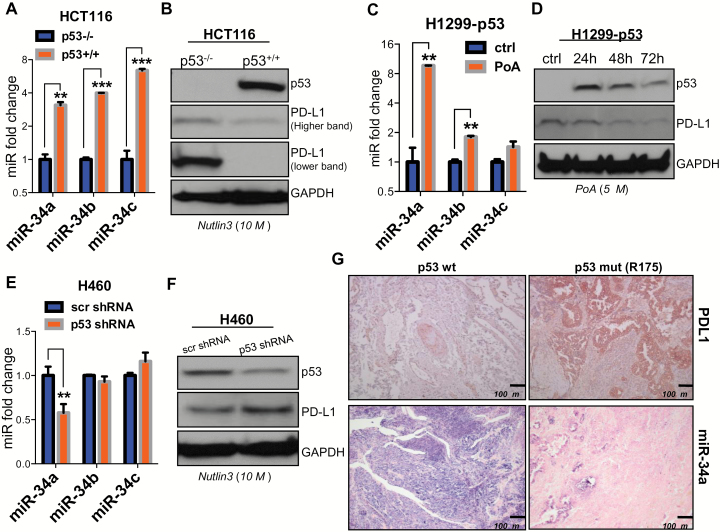
p53 regulation of PDL1 via miR-34. **A**) miR-34a, miR-34b, and miR-34c are upregulated in HCT116 p53^+/+^ cells treated with nutlin 3 (10 µM for 24 hours) (*P* = .0001, *P <* .001, *P <* .001) and in **(C)** H1299 p53-inducible cells treated with ponasterone A (PoA) (5 µM for 24, 48, and 72 hours) compared with their respective controls, confirming that p53 overexpression induces miR-34a, miR-34b, and perhaps miR-34c expression (*P* = .001, *P* = .004, *P* = .09). An unpaired t test was used to calculate the two-sided P values. **E**) Downregulation of miR-34a but not miR-34b or miR-34c, in H460 cells expressing p53-targeting shRNA compared with control (*P* = .006, *P =* .19, *P* = .20). An unpaired *t* test was used to calculate the two-sided *P* values. **B**) Downregulation of PDL1 expression in HCT116 p53^+/+^ vs p53^-/-^ cells and in H1299 p53-inducible cells **(D)** and upregulation of PDL1 in H460 cells treated with p53-shRNA **(F)**. **G**) Immunohistochemical staining of samples from patients with NSCLC showing higher PDL1 protein levels (**top row**) in tumors with p53 mutation than in tumors with wild-type (wt) p53 (3 patients per group). Chromogenic in situ hybridization staining (CISH; **bottom row**) indicated downregulation of miR-34a in tumors with mutated p53 relative to tumors with wt p53. Magnification ×100. **Scale bar** = 100 μm. **Error bars** on the **bar charts** represent standard deviation. *P < .05, **P < .01, ***P < .001. ctrl = control; mut = mutated; PoA = ponasterone A; wt = wild-type.

To investigate the role of p53 in PDL1 regulation, we used three different cell systems and determined whether the specific induction or depletion of p53 affects PDL1 expression: 1) isogenic HCT116 p53^-/-^ and p53^+/+^ colon cancer cells treated with the p53 stabilizer nutlin 3 (26), 2) p53-inducible H1299 lung cancer cells treated with ponasterone A (PoA) (27,28), and 3) H460 lung cancer cells transfected with a p53-specific or a scrambled shRNA. p53 expression or lack thereof was confirmed by western blotting (Figure 1, B, D, and F). Because miR-34 family members are well-characterized effector molecules that are transcriptionally induced by p53 and because p53 regulates tumor cell recognition by natural killer (NK) cells via miR-34a (10), we tested miR-34 family expression in our p53 in vitro models. As expected, miR-34a, miR-34b, and miR-34c were expressed at elevated levels in cells that expressed wild-type p53 (HCT116 p53^+/+^ and p53-inducible H1299 treated with PoA) relative to their controls (HCT116 p53-/- and p53-inducible H1299 in the absence of PoA) (Figure 1, A and C). In the HCT116 colon cancer cells, levels of miR-34b and miR-34c were higher than levels of miR-34a. On the other hand, in H1299-p53 cells induction of miR-34a was higher than induction of miR-34b and miR-34c (*P* = .0001, *P* < .001, *P* < .001 vs *P* = .001, *P* = .004, *P* = .09). In H460+p53 shRNA cells, we noted some downregulation of miR-34b and miR-34c expression relative to H460+scr shRNA, but the decrease was statistically significant only for miR-34a (*P* = .006, *P* = .19, and *P* = .20) (Figure 1E). In contrast, PDL1 was lost or expressed at reduced levels in cells that expressed wild-type p53, suggesting that induction of p53 promoted the downregulation of PDL1 relative to controls (Figure 1, B, D, and F). To confirm this inverse relationship of miR-34a and PDL1 expression in vivo, we used p53-wt and p53-mutated (R175) NSCLC patient samples. As shown in Figure 1G, NSCLC tumors with mutated p53 had low miR-34a and high PDL1 levels compared with tumors with wt p53.

### Correlation Between p53 Expression and PDL1 in TCGA Samples From Patients With NSCLC

**Figure 2.**
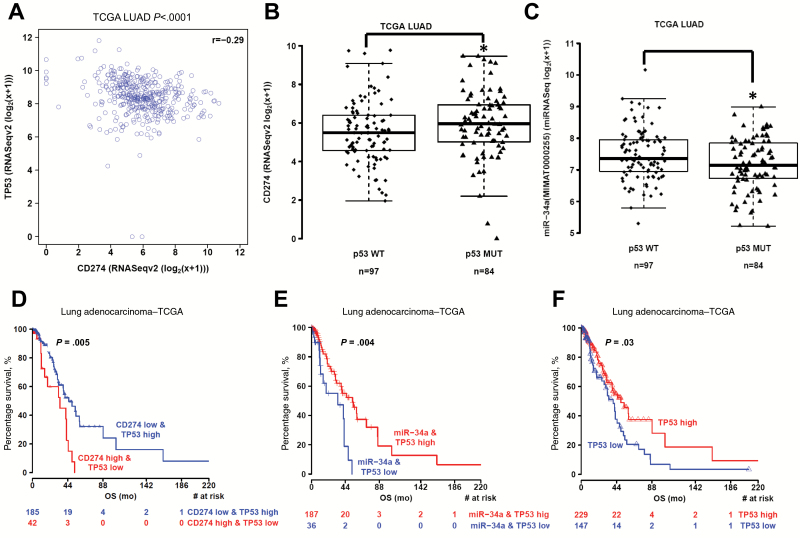
Correlation of p53 with PDL1 expression in patients with non–small cell lung cancer. **A**) Correlation between p53 and PDL1 (CD274) mRNA expression in samples from 181 patients with NSCLC from The Cancer Genome Atlas (TCGA) (*P* < .001) (29). The Spearman’s rank-order correlation test was applied to measure the strength of the association between p53 and PDL1 (CD274) mRNA levels. **B**) PDL1 expression in patients with p53-mutated tumors (n = 84) and p53 wild-type (wt) tumors (n = 97) showed that tumors with mutated p53 had higher PDL1 levels than did those with wt p53 (*P* = .03). CD274 levels were compared between p53 mutant tumors and p53 wt tumors with Mann-Whitney tests. **C**) miR-34a levels were lower in tumors with mutated p53 vs wt p53 (*P* = .01). A **box-and-whisker plot** is used to represent the data. **Box plot** represents first (lower bound) quartile, median and third (upper bound) quartile. **Whiskers**, representing 1.5 times the interquartile range, were used to visualize data (log2) for these comparisons. Mann-Whitney-Wilcoxon test. Two-sided. **D**) Kaplan-Meier overall survival curves according to CD274 and TP53 expression in TCGA LUAD patients cohorts. The number of patients at risk in high CD274/low TP53 and low CD274/high TP53 groups at different time points are presented at the **bottom of the graph**. Log-rank test, two-sided. Patients with tumors that expressed high PDL1 and low p53 levels had lower survival rates than did patients with low PDL1/high p53 tumors (*P* = .005). **E and F**) Patients with high miR-34a/high p53 or high p53-only tumors had better survival rates than did patients with low miR-34a/low p53 tumors **(E)** (*P* = .004) or low p53-only tumors **(F)** (*P* = .03). The log-rank test was used to determine the association between mRNA/miRNA expression and overall survival, and the Kaplan-Meyer method was used to generate survival curves. All tests were two-sided and considered statistically significant at the .05 level. *P < .05, **P < .01, ***P < .001. LUAD = lung adenocarcinoma; TCGA = The Cancer Genome Atlas; WT = wild-type.

We next analyzed the correlation between p53 expression and PDL1 (CD274) in TCGA samples from 181 patients with NSCLC (29). First, we compared the mRNA expression levels of p53 and PDL1, as done in another study (30). We found a statistically significant inverse correlation between p53 and PDL1 (*r* = -0.29, *P* < .001) (Figure 2A). A second analysis comparing PDL1 expression in NSCLC tumors with mutated p53 (n = 84) vs wt p53 (n = 97) revealed that mutated p53 tumors had statistically significantly higher PDL1 levels than wt p53 tumors (*P* = .03) (Figure 2B). Accordingly, miR-34a levels were lower in patients with mutated vs wt p53 (*P* = .01) (Figure 2C). Patients had higher levels of miR-34a than of miR-34* or miR-34bc (Supplementary Figure 1, available online). No differences in miR-34a*, miR-34b, or miR-34c levels were noted in patients with mutated vs wild-type p53 (Supplementary Figure 2, available online). We next stratified patients based on the type of p53 mutation (functional, partially functional, or nonfunctional mutations) by using the IARC TP53 Database (31) (http://p53.iarc.fr/TP53GeneVariations.aspx) and analyzed expression of miR-34a and PDL1 (Supplementary Figure 3, available online). We found no statistically significant differences in PDL1 among the subgroups (*P = .32*), although patients with partially functional or nonfunctional p53 seemed to have higher expression of PDL1 than did patients with wt or functional p53. We did find a marginally statistically significant difference in miR-34a levels among these subgroups (*P* = .046). Notably, only PDL1 mRNA levels were available for analysis; our results would be more conclusive if we could analyze PDL1 protein levels as well. To check for possible relationships between survival and p53, PDL1, and miR-34a, we first grouped the TCGA NSCLC patient data into percentiles according to mRNA/miRNA expression and determined that the best cutoff point for low/high p53 was 0.39. No statistically significant difference was noted for miR-34a, but we did find a borderline statistically significant difference for PDL1 (*P* = .05) at a cutoff of 0.69 (Supplementary Figure 4, available online). We then considered whether combining the expression of both factors (p53 and PDL1) would improve separation between groups. For the p53/PDL1 pair, we contrasted the two groups linked to a negative association, high p53/low PDL1 and low p53/high PDL1, and found that the best separation occurred at a cutoff of 0.69 for PDL1 and 0.28 for p53 (*P* = .005) (Figure 2D). The difference in survival between these two groups was strikingly greater than the difference between groups based on p53 expression alone (*P* = .03) (Figure 2F). For the p53/miR-34a pair, we contrasted the two groups linked to a positive association: high p53/high miR-34a and low p53/low miR-34a. The best separation was obtained for miR-34a at a cutoff of 0.32 and for p53 at a cutoff of 0.28 (*P* = .004) (Figure 2E). This result was also an improvement over the results obtained for p53 alone (Figure 2F). Next, we randomly divided the available set of samples into two halves, a training set and a validation set, and we applied the procedure described above to each set. We validated the result obtained for TP53 (cutoff = 0.31) and miR-34a (cutoff = 0.33) (Supplementary Figure 5, available online). No information on tumor grade was available via TCGA portal, but a multivariable regression analysis adjusted for disease stage showed that low expression of miR-34a and p53 independently predict poor prognosis (Supplementary Table 1, available online).

### miR-34 Family Regulation of PDL1 in NSCLC Cell Lines

**Figure 3.**
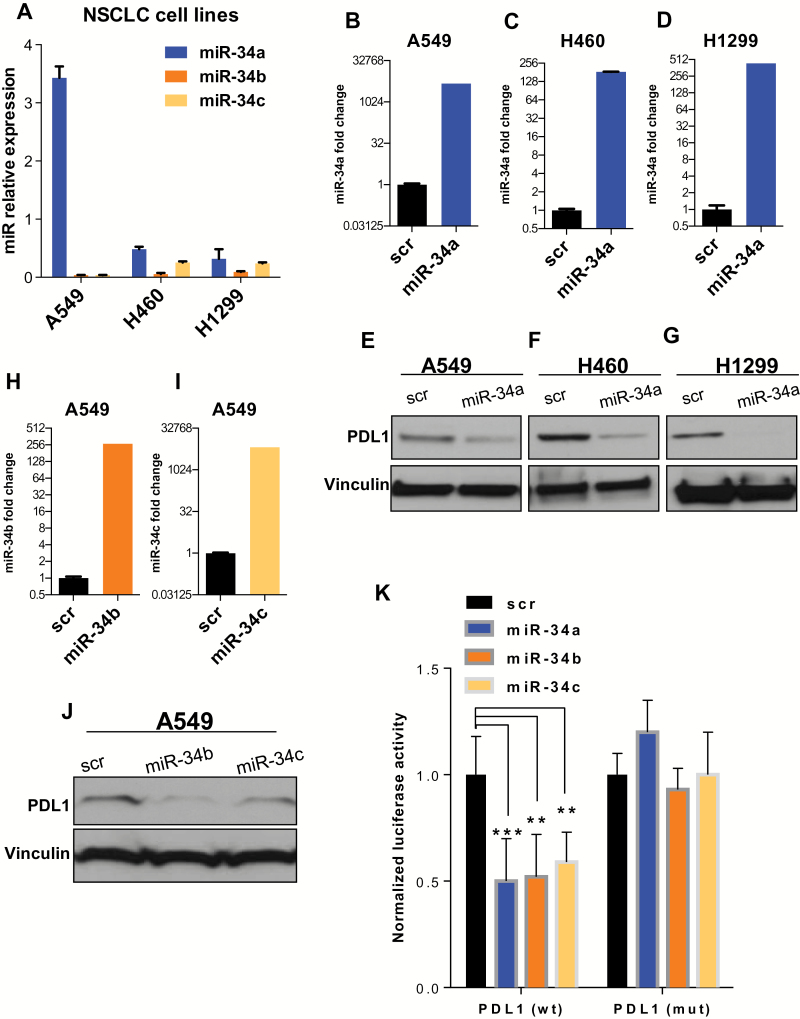
miR-34s regulation of PDL1. Endogenous levels of miR-34a, -b, and -c in non–small cell lung cancer (NSCLC) cell lines. **A**) PDL1 expression in A549, H460, and H1299 cells transfected with miR-34a, -b, and -c. **B-J**) NSCLC cell lines were treated with miR-34a, miR-34b, or miR-34c at 100nM, and 24 hours later RNA was isolated to study miR-34a, -b, and -c transfection efficiency. At 96 hours after transfection, cell lysates were collected for protein analysis. Quantification of western blots shows that forced overexpression of miR-34a, miR-34b, or miR-34c suppressed PDL1 protein expression compared with a scrambled control. **K**) Luciferase activity in cells cotransfected with miR-34a, -b, or -c or a scrambled control and a luciferase reporter construct encoding the luciferase gene fused either to the wild-type *PDL1* 3’ UTR (PDL1 wt) or a mutated *PDL1* 3’ UTR (PDL1 mut). All three of the miR-34s reduced luciferase activity (*P < .*001, *P* =.006, and *P* =.006). An unpaired *t* test was used to calculate the two-sided *P* values. *P < .05, **P < .01, ***P < .001. **Error bars** on the **bar charts** represent standard deviation. Similar results were observed in three replicates. mut = mutated; NSCLC = non–small cell lung cancer; scr = scrambled; wt = wild-type.

The inverse correlation of miR-34 and PDL1 expression in NSCLC cells and in tumor samples implicated miR-34 as a regulator downstream of p53 to repress PDL1. This hypothesis was further corroborated by the observation that the 3’ untranslated region (UTR) of the *PDL1* mRNA carries a putative miR-34 binding site at position 932–938 (32,33). We first analyzed endogenous levels of miR-34a, -b, and -c in NSCLC cell lines with different genetic backgrounds (Figure 3A). In agreement with our findings in NSCLC patient samples (Supplementary Figure 1, available online), miR-34a expression levels were higher than miR-34b and miR-34c levels in all three NSCLC cell lines. As shown in Figure 3, B-G, enforced overexpression of miR-34a suppressed the expression of PDL1 protein compared with a scrambled control. In addition, enforced overexpression of miR-34b or miR-34c suppressed the expression of PDL1 protein compared with a scrambled control (Figure 3, H-J). Next, to determine whether miR-34 interacts directly with the putative target gene *PDL1*, we cotransfected H1299 cells with miR-34a, -b, or -c mimics and a reporter vector encoding the luciferase gene that is fused to the 3′ UTR of the *PDL1* gene (luc-PDL1). As shown in Figure 3K, luciferase activity was reduced in cells transfected with miR-34 and the luc-PDL1 construct compared with scrambled controls. In contrast, mutation of the predicted miR-34 binding site in the 3’ UTR of *PDL1* rescued the luciferase activity, thus confirming that miR-34a, miR-34b, and miR-34c interact directly with the PDL1 3′ UTR (Figure 3K) (fold-change luciferase activity, miR-34a mean = 0.50, SD = 0.2, *P < .*001; miR-34b mean = 0.52, SD = 0.2, *P* = .006; and miR-34c mean = 0.59, SD = 0.14, *P* = .006).

### PDL1 Expression After In Vivo Delivery of miR-34a

**Figure 4.**
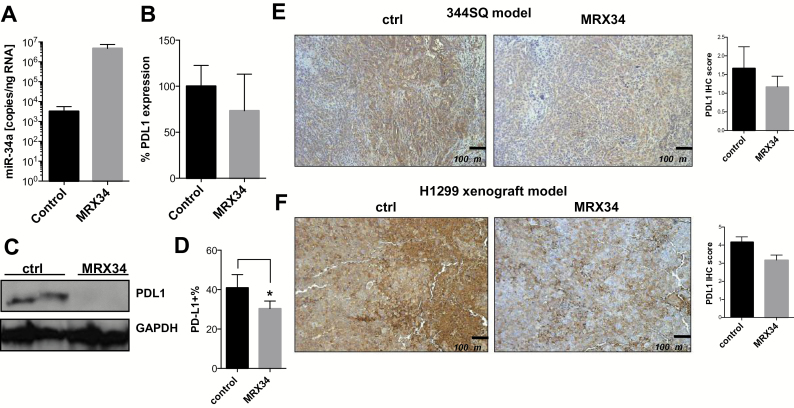
PDL1 expression after in vivo delivery of miR-34a. Analysis of miR-34a and PDL1 expression levels in subcutaneous 344SQ tumors treated with MRX34 (n = 2) by quantitative polymerase chain reaction **(A)**, by western blotting **(C)**, by flow cytometry (**B**) (*P* = .04, n = 2; an unpaired *t* test was used to calculate the two-sided *P* value) **(D),** or by immunohistochemical staining **(E)** in a syngeneic mouse model. **Scale bar** = 200 μm. **F**) MRX34-induced downregulation of PDL1 in an H1299 xenograft model described elsewhere (37). Magnification ×100. **Scale bar** = 100 μm. *P < .05. **Error bars** on the **bar charts** represent standard deviation. ctrl = control; IHC = immunohistochemistry; MRX34 = miR-34a–loaded liposomes.

Next, we tested the effects of miR-34a replacement on PDL1 expression in a syngeneic mouse model of NSCLC. To this end, we peritumorally administered MRX34, a liposomal nanoparticle loaded with miR-34a mimics, to murine 344SQ tumors grown subcutaneously in mice (25). We observed that MRX34 treatment increased miR-34a levels in tumors and concurrently downregulated tumor *PDL1* mRNA and PDL1 protein as measured by quantitative real-time polymerase chain reaction and western blotting (Figure 4, A-C). One explanation for the differences found on mRNA and protein levels may be related to the fact that some miRNAs can repress translation of their mRNAs targets, with little or no influence on their abundance (34,35). Although protein expression can be inhibited by miRNAs, mRNAs can be detected in polysomes, suggesting that they can be repressed after translation has begun (34–36). The miR-34–induced repression of PDL1 was further confirmed by flow cytometry (mean of PDL1 expression percentage of control group = 42.9%, SD = 8.1%; mean of PDL1 expression percentage of MRX34 = 30.4%, SD = 3.8%, *P* = .04, n = 2) (Figure 4D) and immunohistochemical staining of 344SQ tumor tissue (Figure 4E). Liposomal delivery of miR-34 mimics also repressed PDL1 in subcutaneous H1299 NSCLC xenografts (37) (Figure 4F). In agreement with the general observation that miRNAs typically repress a given target by 30% to 70%, our in vitro findings showed that maximum exposure to the miR-34 mimic repressed expression of the luciferase reporter (fused to the PDL1 3’ UTR) by approximately 50% (Figure 3K). In light of these data and under conditions of optimum miR-34 delivery, we would expect a similar level of repression in vivo. In fact, quantification of both western blot and immunohistochemical findings (344SQ and H1299 models) demonstrated that liposomal delivery of miR-34 in vivo repressed PDL1 by approximately 30%. To explain this apparent discrepancy, Stalder et al. (38) showed that only 10% to 25% of injected siRNA material has the potential to be loaded into RNA-induced silencing complexes and that only a fraction of that is therapeutically active. Our analysis of miR-34 concentrations in the tumor tissue does not distinguish between active and inactive miRNA, and most of what we quantify is presumably extracellular and therefore inactive.

### Impact of In Vivo Delivery of miR-34a, Alone or in Combination With XRT, on Immune Cell Populations in the Tumor Microenvironment

**Figure 5.**
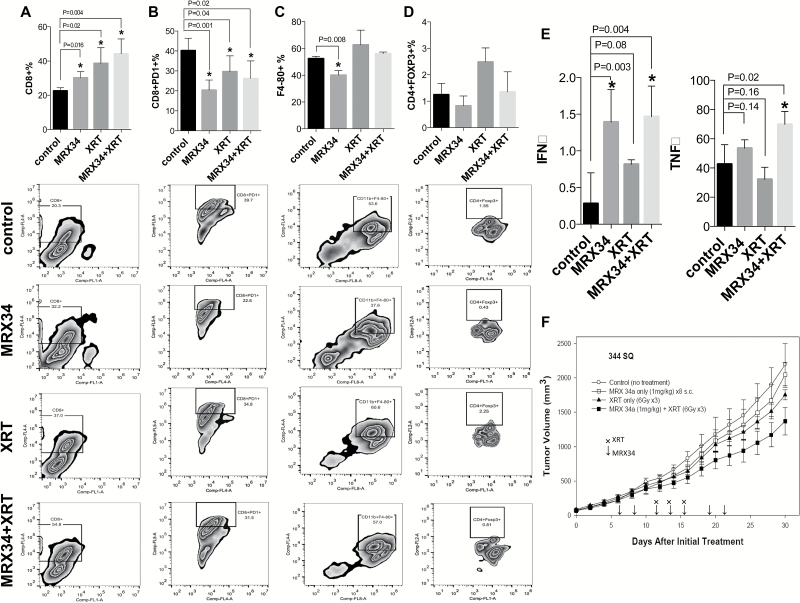
Impact of therapeutic miR-34a delivery combined with radiotherapy on immune cell populations in the tumor microenvironment. Subcutaneous tumors were created by inoculating 1 x 10^6^ 344SQ cells derived from a spontaneous subcutaneous lung metastasis from a p53R172H^Δg/+^K-ras^LA1/+^ mouse (25) into the right leg of each syngeneic 129Sv/Ev mouse. One week after tumor implantation, mice were randomly assigned to one of four groups: control, MRX34 only, radiation (XRT), or MRX34 plus XRT. The MRX formulation was given as subcutaneous injections at a dose of 1mg/kg (total of 8 injections), and local irradiation was given in 6-Gy fractions to a total dose of 18 Gy over three days, starting when the tumors were 8mm in diameter. For the combination-therapy condition, MRX34 was given one hour before XRT. **A**) One week after treatment completion, flow cytometry revealed that MRX34 + XRT increased the number of CD8+ cells compared with control or either treatment given alone (*P =* .004, n = 4). **B and C**) MRX34 reduced the numbers of PD1+ T-cells **(B)** and macrophages **(C)** (*P* = .001 and *P* = .008, n = 4). MRX34+XRT combination treatment was more effective in reducing the numbers of PD1+ T-cells (*P* = .02, n = 4) and macrophages than was XRT alone. (**d**) XRT seemed to increase the numbers of T-regulatory cells (Tregs) over the control condition, but none of the treatments showed statistically significant effects on Tregs. **E**) Interferon-gamma levels were increased by MRX34 only and by MRX34 + XRT vs control or XRT alone; MRX34 + XRT increased levels of tumor necrosis factor–alpha. An unpaired *t* test was used to calculate the two-sided *P* values. **F**) MRX34 + XRT delayed tumor growth compared with the control condition in a 344SQ mouse model (n = 6). *P < .05. **Error bars** on the **bar charts** represent standard deviation. IFNγ = interferon-gamma; MRX34 = miR-34a–loaded liposomes; TNFα = tumor necrosis factor–alpha; XRT = radiotherapy.

To explore the effects of MRX34 on tumor growth, the tumor milieu, and its associated immune cells, we designed a multidose efficacy study in the 344SQ syngeneic mouse model. One week after subcutaneous inoculation of the 344SQ xenograft, mice were randomly assigned to a MRX34 or a control group. Because XRT has been shown to induce adaptive immune responses that promote tumor regression (39), we also tested the effects of MRX34 in combination with XRT and assigned mice to a combination group and an XRT-only group. MRX34 treatment resulted in increased numbers of tumor-infiltrating CD8+ cells (mean of CD8 expression percentage of control group = 22.5%, SD = 1.9%; mean of CD8 expression percentage of MRX34 = 30.1%, SD = 3.7%, *P* = .02, n = 4) (Figure 5A) and reduced the numbers of tumor-infiltrating PD1+ T-cells (mean of CD8/PD1 expression percentage of control group = 40.2%, SD = 6.2%; mean of CD8/PD1 expression percentage of MRX34 = 20.3%, SD = 5.1%, *P* = .001, n = 4) (Figure 5B), macrophages (mean of F4-80 expression percentage of control group = 52.4%, SD = 1.7%; mean of F4-80 expression percentage of MRX34 = 40.1%, SD = 3.5%, *P* = .008, n = 4) (Figure 5C), and perhaps T-regulatory cells (Tregs) (Figure 5D) compared with the control condition. XRT alone also increased CD8+ cells (mean of CD8 expression percentage of XRT to control group = 38.6%, SD = 9.2%, *P* = .02, n = 4) (Figure 5A) and decreased PD1+ cells in the tumor (mean of CD8/PD1 expression percentage of XRT to control group = 29.6%, SD = 7.9%, *P* = .04, n = 4) (Figure 5B); however, and in contrast to MRX34, XRT led to an increase of macrophages and Tregs (Figure 5, C and D). The combined use of MRX34 and XRT resulted in an even greater increase in CD8+ T-cells (mean of CD8 expression percentage of MRX34 plus XRT to control group = 44.2%, SD = 8.7%, *P* = .004, n = 4) (Figure 5A) compared with each therapy alone and decreased PD1+ T-cells (mean of CD8/PD1 expression percentage of MRX34 plus XRT to control group = 26.1%, SD = 8.9%, *P* = .02, n = 4) (Figure 5B). MRX34 in combination with XRT also counteracted the effects of XRT on macrophages and Tregs, both of which were lower in the combination condition relative to XRT alone. These treatments, alone and in combination, did not affect numbers of dendritic cells and may have slightly increased the numbers of MDSCs (Supplementary Figure 6, available online). In accordance with the decline in exhausted T-cells (PD1+) and the increase in CD8^+^ T-cells, we found that MRX34 and XRT produced increases in interferon-gamma (IFNγ) (mean of IFNγ levels of control group = 0.28 pg/mL, SD = 0.42 pg/mL; mean of IFNγ levels of MRX34 plus XRT = 1.5 pg/mL, SD = 0.41 pg/mL, *P* = .004, n = 4) and in tumor necrosis factor–alpha (TNFα) (mean of TNFα levels of control group = 42.52 pg/mL, SD = 13.45 pg/mL; mean of TNFα levels of MRX34 plus XRT = 69.78 pg/mL, SD = 8.97 pg/mL, *P* = .02, n = 4) compared with the control conditions; IFNγ levels were also increased in the MXR34-only condition (mean of IFNγ levels of MRX34 = 1.4 pg/mL, SD = 0.44 pg/mL, *P* = .003, n = 4) (Figure 5E). Finally, MRX34, XRT, and MRX34 plus XRT all delayed tumor growth relative to the control group (n = 6), with the MRX34+XRT combination being the most effective (Figure 5F).

## Discussion

Previous studies have shown that p53 interacts with the immune response by regulating inflammatory cytokines (5), toll-like receptors (6,7), and IFN signaling (8,9) and by modulating activation of T-cells and NK cells (40). However, the involvement of p53 in tumor immune evasion is poorly understood. Our findings from this study define a new role for p53 and suggest that p53 specifically modulates the tumor immune response by regulating PDL1 via miR-34. This ties tumor immune evasion to other tumor suppressor pathways previously described for p53 and miR-34a, such as apoptosis, DNA damage, and cell cycling (41–43), and further complements recent findings implicating p53 and miR-34 in immune cell regulation. For instance, p53 regulates tumor cell recognition by NK cells via miR-34a regulation (10). miR-34 can also function in a feedback loop to tumor growth factor-beta (TGFβ), regulating the chemokine CCL22 and tumor immune escape via recruitment of Tregs (44). Another study showed that miR-34a regulates diacylglycerol kinase ζ (DGKζ), a protein that regulates T-cell activation after engagement of the T-cell receptor (45). During preparation of this manuscript, it was reported that miR-34a targets PDL1 in acute myeloid leukemia (46). Thus, p53 modulates several aspects of tumor immunity, and some of these aspects seem to be controlled by miRNAs that operate downstream of p53. Evidence to support this is also provided by miR-200, an miRNA that is also controlled by p53 and directly represses PDL1 (14).

Our findings that patients with NSCLC that expressed high *PDL1* and low *p53* levels had lower survival rates than did patients with low *PDL1*/high *p53* tumors and those with high miR-34a/high *p53* had better survival rates than those with low miR-34a/low *p53* or simply low *p53* expression have potential clinical applications because the concomitant status of p53 and PDL1 expression could be useful biomarkers of response to therapy. Further study will be needed to confirm this supposition before p53 and PDL1 expression can be used as biomarkers in the clinic.

Previous studies have suggested that PD1/PDL1 signaling induces T-cell exhaustion, a process described broadly as dysfunction and subsequent physical deletion of specific T-cells and more specifically as changed intratumoral levels of CD8^+^ T-cells, macrophages, MDSCs, and dendritic cells (47). T-cell exhaustion is accompanied by progressive decreases in the production of cytokines such as IFNγ, TNFα, and interleukin-2 (IL2) (47). Consequently, PD1/PDL1 blockade can restore function in some subtypes of exhausted CD8^+^ T-cells (48–50) and lead to an antitumor immune response via regulation of several aspects of the tumor microenvironment (51–54). In line with this phenotype, the in vivo delivery of miR-34 via MRX34 in our syngeneic tumor model increased the number of tumor-infiltrating CD8^+^ T-cells and decreased the number of exhausted CD8+PD1+ T-cells, macrophages, and Tregs, suggesting that miR-34 may have a direct effect on immune evasion that can be exploited therapeutically. The effect on CD8^+^ T-cells was augmented in combination with XRT, which has also been shown to induce adaptive immune responses (39). This result also agrees with another study showing that miR-34a promotes T-cell activation by regulating DGKζ and CD69 (45). Interestingly, we further found that MRX34 decreased PD1^+^ T-cells compared with control, XRT-only, or combination therapy. In accordance with the decreased numbers of PD1^+^ cells, we also found that IFNγ and TNFα were increased by MRX34 and XRT. These results indicate that miR-34a has a key role in T-cell exhaustion. Here, we uncovered a new function of miR-34 and now demonstrate that miR-34 can also reactivate the immune system in fully immunocompetent mice. Our findings on macrophages and Tregs agree with those of previous studies showing that miR-34a regulates CSF1R, the receptor for CSF1 expressed by macrophages (55,56).

A limitation of this study is the fact that the differences on the tumor microenviroment between xenografts and orthotopic in vivo models might influence immune cell profiling. Studies are in progress to specifically address this issue. In addition, other studies are necessary to determine if PDL1 expression or tumor-infiltrating T-cells mediate an impact on tumor growth after miR-34 delivery and to distinguish the effects of miR-34a on oncogenic pathways from that affecting tumor immune evasion.

Taken together, our findings identify a novel mechanism by which tumor immune evasion is regulated by p53 and miR-34a via PDL1. The results further suggest that therapeutic delivery of miR-34a combined with standard therapies, such as XRT, may represent a new modality of immunotherapy.

## Funding

This work was supported by a Doctors Cancer Foundation Grant, The Lung Cancer Research Foundation, Cancer Center Support (Core) Grant CA016672 to The University of Texas MD Anderson Cancer Center, the Mabuchi Research fund, the family of M. Adnan Hamed, the Susan and Peter Goodwin Foundation, and the Orr Family Foundation to MD Anderson Cancer Center’s Thoracic Radiation Oncology program, an MD Anderson Knowledge Gap award, the Department of Defense (BATTLE award W81XWH-06-1-0303 and PROSPECT award W81XWH-07-1-03060), and the Wiegand Foundation. HJP, KK, DM, and AGB were supported by a commercialization grant from the Cancer Prevention Research Institute of Texas (CPRIT). GAC is a Alan M. Gewirtz Leukemia & Lymphoma Society Scholar and is supported in part by the NIH/NCI grants 1UH2TR00943-01 and 1 R01 CA182905-01.

## Supplementary Material

Supplementary Data

## Notes

The authors thank Christine F Wogan, MS, ELS, of MD Anderson’s Division of Radiation Oncology, for editorial contributions.

The authors were fully responsible for the design of the study, analysis and interpretation of results, the decision to submit the manuscript, and the writing of the manuscript.

HJP, KK, DM, and AGB are employees of Mirna Therapeutics, which develops miRNA-based therapies. All other authors declare no competing financial interests.
