# Supplementary material for: PDL1 Regulation by p53 via miR-34
Source: J Natl Cancer Inst. 2015 Nov 17;108(1):djv303. doi: 10.1093/jnci/djv303 (PMC4862407; doi:10.1093/jnci/djv303)
Supplement: Supplementary Data [file supp_108_1_djv303__index.html]

Supplementary Data 

# PDL1 Regulation by p53 via miR-34

## Supplementary Data

Data files

- Supplementary Data - Supplementary Data
