# Supplementary material for: PDL1 Regulation by p53 via miR-34
Source: J Natl Cancer Inst. 2015 Nov 17;108(1):djv303. doi: 10.1093/jnci/djv303 (PMC4862407; doi:10.1093/jnci/djv303)
Supplement: Supplementary Data [file supp_djv303_14_0887R2_Welsh_supp_mat_final.docx]

**Supplementary Methods**

**Cell Lines**

Lung cancer cell lines A549, H460, H1299 were obtained from the American Type Culture Collection (ATCC; Manassas, VA, USA) and validated by MD Anderson's Characterized Cell Line Core facility by short-tandem-repeat (STR) DNA fingerprinting with an AmpFlSTR Identifiler PCR Amplification kit (Applied Biosystems #4322288) according to the manufacturer's recommendations. H1299-p53 encoding inducible *TP53* ([25](#_ENREF_1), [2](#_ENREF_2)6) were kindly provided by Dr. Raymond E. Meyn (MD Anderson Cancer Center); HCT116 p53^-/-^ and HCT116 p53^+/+^ cells, by Dr. Bert Vogelstein ([27](#_ENREF_3)) (Johns Hopkins Medical Institute); and 344SQ cells, by Dr. Jonathan Kurie (MD Anderson Cancer Center). Cells were cultured in RPMI supplemented with 10% fetal bovine serum at 37°C in a humidified 5% CO_2_ incubator. HCT116 p53^-/-^ and HCT116 p53^+/+^ and H460 p53-knockdown cells were treated for 24 hours with 10 μM nutlin 3 (Sigma) to stabilize p53 via MDM2 inhibition. To induce p53 expression in H1299-p53 cells, H1299-p53 cells were treated with 5 μM ponasterona A (Sigma).

**Establishment of Stable p53–Knockdown Cells**

The vectors pRS-shRNAp53 and an shRNA scramble control (from Open Biosystems GE Healthcare Dharmacon Inc) were transiently transfected with a pPACKH1 HIV Lentivector Packaging Kit (System Biosciences) into 293TN cells using Lipofectamine 2000 and Plus reagents (both from Life Technologies, Carlsbad, CA, USA). Viral supernatant was collected 3 days after transfection and mixed with PEG-it Virus Precipitation Solution (System Biosciences) overnight at 4°C. H460 cells were infected and incubated with the viral particles supplemented with TransDux (System Biosciences) overnight at 37°C. Puromycin (1 µg/mL) was used to select and maintain p53-knockdown in H460 cells. Stable repression of p53 was verified by western blotting.

**Transfection**

Pre-miR-34 family and negative controls (scrambled oligos) (Life Technologies) were reverse-transfected into lung cancer cell lines with Lipofectamine 2000 (Life Technologies) at a final concentration of 100 nM.

**Quantitative Polymerase Chain Reaction**

Total RNA was isolated from cells with Triazol (Life Technologies) for miRNA analysis according to the manufacturer’s protocol. To analyze expression of mature miR-34 family, total RNA was reverse-transcribed using miRNA-specific primers and the TaqMan MicroRNA Reverse Transcription kit from Life Technologies, followed by quantitative polymerase chain reaction (qPCR) with Taqman MicroRNA assays according to the manufacturer’s protocol. Relative abundance of miRNA versus U6 expression was calculated by the comparative Ct method using Excel softwares (Microsoft Corp). A (two-sided) t-test was applied to compare the mean between different treatments. Analyses were carried out in GraphPad (GraphPad Prism, USA). The statistical significance was defined as a *P* value <0.05.

**Protein Extraction and Western Blot Analysis**

Total protein was extracted as previously described ([35](#_ENREF_4)). Membranes were probed with primary antibodies directed against PDL1 (Pierce-Thermo Fisher Scientific, Rockford, IL, USA), p53, GAPDH (Cell Signaling Technologies, Beverly, MA, USA), and a secondary antibody conjugated with horseradish peroxidase (Amersham GE Healthcare). The secondary antibody was visualized by using a chemiluminescent reagent (Pierce ECL kit, Thermo Fisher Scientific, Waltham, MA, USA).

**Luciferase Assay**

H1299 cells were seeded into 96-well dishes at 4 × 10^4^ cells/well. Cells were transfected with miR-34 family mimics or a scrambled control miRNA (100 nM) together with expression vectors encoding the luciferase gene fused to the *PDL1* 3´ UTR that is either wild type (wt) or contains a mutated miR-34 binding site (5-bp deletion; SwitchGear Genomics, Carlsbad, CA, USA). Forty-eight hours after transfection, cells were incubated for 30 min with 100 µL/well of LightSwitch Luciferase Assay Reagent (SwitchGear Genomics). Firefly luciferase activity was measured sequentially in luciferase assays (SwitchGear Genomics) using a Fluostar Optima plate reader (BMG Lab Technologies GmbH, Inc, Durham, NC, USA). Three independent experiments were performed, and values are shown as means ± standard error of the mean. A (two-sided) t-test was applied to compare the means between different treatments. Analyses were carried out in GraphPad (GraphPad Prism, USA). The statistical significance was defined as a *P* value <0.05.

**Chromogenic *In Situ* Hybridization for miR-34a**

Formalin-fixed paraffin-embedded (FFPE) NSCLC tissue samples, 4–5 μm thick, from patients were mounted on Histogrip-treated microscope slides, dried at 37°C, and baked for 2–4 hours at 60°C. The slides were deparaffinized at 80°C for 30 minutes and then hydrated by dipping the slides into xylene, ethanol and phophate buffered saline (PBS).  A 15 µg/mL solution of pre-warmed (37°C) proteinase-K  (miRCURY LNA microRNA ISH Buffer Set; Exiqon; cat# 90000) was applied and slides were incubated at 37°C for 20 minutes. Then, slides were dehydrated and dried.  Twenty-five microliters of 40 nM denatured double-DIG-labeled miRCURY miR-34a LNA probe (Exiqon; cat# 38487-15) diluted in 1X ISH Buffer (miRCURY LNA microRNA ISH Buffer Set; Exiqon; cat# 90000)  was added to the center of each sample along with coverslips, and slides were incubated at 53°C for 1 hour.  Specificity washes were performed for 5 minutes each at 53°C using 5 x SSC, 1 x SSC, 1 x SSC, 0.2 x SSC, 0.2 x SSC plus one wash with 0.2 x SSC at room temperature (Invitrogen, cat# 15575-038). Tissues were blocked for 15 minutes and incubated for 1 hour at room temperature with anti-DIG antibody alkaline phosphatase (AP) conjugate in a 1:1600 dilution (Roche Life Sciences, cat# 11093274910). After slides were rinsed in PBS-Tween20, freshly prepared AP substrate (NBT/BCIP; Roche Life Sciences) was applied.  Slides were incubated for 2 hours at 30°C, protected from light. Tissue slides were rinsed in KTBT (50 mM Tris-HCl, 150 mM NaCl, 10 mM KCl) and nuclease-free water and counterstained with fast red counter stain (American Master Tech; cat# STNFR).

***In Vivo* Administration of miR-34a/Liposome Complexes**

When tumors reached a volume of approximately 95 mm^3^ (range 50–139 mm^3^), mice were randomly assigned to one of the following groups such that each group had the same average tumor volume (12 mice per group): Control (no treatment); MRX34 (administered peritumorally three times a week at a dose level of 1 mg per kg of mouse body weight for a total of 8 injections; Mirna Therapeutics, Austin, TX ([22](#_ENREF_5), 2[6](#_ENREF_6))); XRT (6 Gy once a day for 3 days, total 18 Gy); or MRX34 + XRT (dose levels and regimens as described above; MRX34 was given 1-2 hours before XRT). Mice were immobilized in a jig, and tumors centered within a 3-cm-diameter circle were irradiated with a ^137^Cesium device (dose rate 4 Gy/min). Tumor growth was assessed 2‒3 times a week by measuring tumor volumes using digital calipers and the formula V = length × width^2^/2, where length is greater than width. Twenty-four hours after the final injection of MRX34, two mice per group were killed and tumor tissues were collected for immunohistochemical and RNA/protein analyses to evaluate miR-34a and PDL1 mRNA and protein expression levels. Total RNA extraction and qRT-PCR were done as previously described (27). For PDL1 expression, we used specific Taqman probes (Life Technology).

**Immunohistochemical Analysis of PDL1**

Formalin-fixed patient samples and mouse tissues were processed in an automatic tissue processor, embedded in paraffin (Peloris, Leica) and cut into 4-μm sections. Immunohistochemical staining was done in an automated staining system (Leica Bond Max, Leica Microsystems, Vista, CA, USA). Briefly, slides were deparaffinized and hydrated and antigen was retrieved by incubating in citrate buffer, pH 6.0 for 1 hour with PDL1 (Thermo Fisher Scientific, #PA5-20343, dilution 1:100). Staining was microscopically evaluated and graded based on percentage of PDL1-positive cells as follows: 0=undetectable, 1=1%-25%, 2=26%-50%, 3=51%-75% and 4=76%-100%. The intensity of cellular staining was also graded as undetectable (0), minimal (1), mild (2), moderate (3) or marked (4).

**Isolation of Tumor-Infiltrating T Cells, Macrophages, Dendritic Cells, and Myeloid-Derived Suppressor Cells**

Twenty-four hours after the last injection of MRX34, freshly isolated primary tumor tissues (4 mice/group) were washed with ice-cold PBS and digested in PBS supplemented with 3 mg/mL dispase II and 2 mg/mL collagenase at 37°C for 1 hour. Single-cell suspensions were prepared by filtering the digested tissues through 70‐μm-pore cell strainers; erythrocytes were removed with red blood cell lysis buffer, and then single-cell suspensions were used for surface or intracellular flow cytometry staining. CD45 staining was used to distinguish immune cells from non-immune cells (tumor and other stromal cells) in tumor tissues, and cells quantified were CD8^+^ T cells, CD4^+^ T cells, macrophages (F4/80), myeloid-derived suppressor cells (MDSCs) (CD11b^+^Gr1^+^) and T-regulatory T cells (Tregs) (CD4^+^CD25^+^Foxp3^+^). Concomitantly, we collected tumor cells for PDL1 analysis. Samples were analyzed with an LSRII Flow Cytometer (BD) and data analyzed with FlowJo Software (ThreeStar). Isotype control was used as negative control. The expression of each marker in an untreated control group was considered the basal expression level. A (two-sided) *t*-test was applied to compare the mean between different treatments. Analyses were carried out in GraphPad (GraphPad Prism, USA). Statistical significance was defined as a *P* value <0.05.

**Assays for TNFα and IFNγ**

Twenty-four hours after the last injection of MRX34, whole blood samples were collected by cardiac puncture and mixed in EDTA coating tubes from control, MRX34, RT and MRX34+RT treatment groups (4 mice/group). The samples were centrifuged at 1,000 *g* for 10 min. Serum was collected and diluted 1:4 with diluent solution from BioPlex Multiplex assay (Biorad). TNFα and IFNγ were measured by ELISA according to the manufacturer’s protocol (Biorad). A (two-sided) *t*-test was applied to compare the mean between different treatments. Analyses were carried out in GraphPad (GraphPad Prism, USA). Statistical significance was defined as a *P* value <0.05.

**Analysis of TCGA data**

Level 3 Illumina RNASeqv2 and miRNASeq were used to analyze mRNA and miRNA expression, respectively. For the miRNASeq data, “reads_per_million_miRNA_mapped” values were derived from the “isoform_quantification” files to calculate mature forms for each miRNA. Somatic mutation data from the Lung adenocarcinoma (LUAD) samples were downloaded from cBioPortal. Analyses were done in R (version 3.0.1) (http:///www.r-project.org/). All tests were two-sided and considered significant at the 0.05 level. CD274 levels were compared between p53 mutant tumors and p53 wt tumors with Mann–Whitney tests, as miR-34a levels were not normally distributed (*P*<0.05 from the Shapiro Wilk normality test). miR-34a levels were compared between p53 mutant tumors and p53 wt tumors with *t* tests, as miR-34a levels were normally distributed (*P*>0.05 from the Shapiro Wilk normality test). A box-and-whisker plot (in which the box plot represents the first (lower bound) and the third (upper bound) quartiles, and the whiskers represent 1.5 times the interquartile range) was used to visualize data (log2) for these comparisons. For each gene/miRNA of interest, we checked for a relation with the survival as follows. Patients were grouped into percentiles according to mRNA/miRNA expression. The log-rank test was used to determine the association between mRNA/miRNA expression and overall survival and the Kaplan-Meyer method was used to generate survival curves. *P* values were obtained for the cut-off to optimally separate the patients in high/low (min *P* value) were recorded. We then considered whether adding second expression level added information. We used the following procedure. A fixed cut-off for gene1 together with a fixed cut-off for gene2 split the cohort in four groups corresponding to low/high gene1 and low/high gene2 expression. For TP53/CD274 we contrasted for each pair cut-offs the two groups linked to a negative association: tumors with high levels of TP53 and low levels of CD274 versus tumors with low levels of TP53 and high levels of CD274. For TP53/miR-34a, we contrasted for each pair of cut-offs the two groups linked to a positive association: tumors with high levels of TP53 and high levels of miR-34a versus tumors with low levels of TP53 and low levels of miR-34a. We recorded the best separation obtained (min *P* value) for each pair and noticed that the difference in median survival time between the two groups contrasted for the pair TP53/CD274 as well for the pair TP53/miR-34a is significantly larger than the difference between the groups classified into high/low based on the expression of TP53 alone.

**Supplementary Table 1.** Multivariable regression analysis adjusted for stage of patients from the TCGA LUAD.*

| **Variable** | **UNIVARIATE ANALYSIS** | | **MULTIVARIATE ANALYSIS** | |
| --- | --- | --- | --- | --- |
|  | **HR (95% CI)** | ***P* Value (log-rank)** | **HR (95% CI)** | ***P* Value (Wald)** |
| Pathologic stage (III-IV vs I-II) | 2.98 (1.68, 5.29) | <.001 | 2.69 (1.5, 4.82) | <.001 |
| miR-34a high & TP53 high vs miR-34 low & TP53 low | 0.38 (0.21, 0.72) | <.001 | 0.45 (0.24,0.85) | 0.01 |

*****All statistical tests were two-sided. CI=confidence interval; HR=hazard ratio; LUAD= Lung adenocarcinoma; TCGA=The Cancer Genome Atlas.

**Supplementary Figure 1.** Expression of miR-34a, -b, and -c in patients from The Cancer Genome Atlas for Lung Adenocarcinoma (TCGA LUAD). Box plot represents first (lower bound) quartile, median and third (upper bound) quartile. Whiskers represent 1.5 times the interquartile range. miRNA Seq= microRNA sequencing (TCGA).


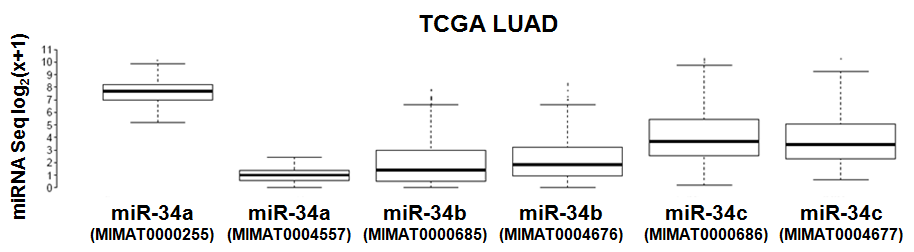


**Supplementary Figure 2.** Expression of miR-34a, -b, and -c according to p53 mutation status in patients from The Cancer Genome Atlas for Lung Adenocarcinoma (TCGA LUAD). Box plot represents first (lower bound) quartile, median and third (upper bound) quartile, whiskers represent 1.5 times the interquartile range. Mann–Whitney–Wilcoxon test. Two-sided. miRNA Seq= microRNA sequencing (TCGA).


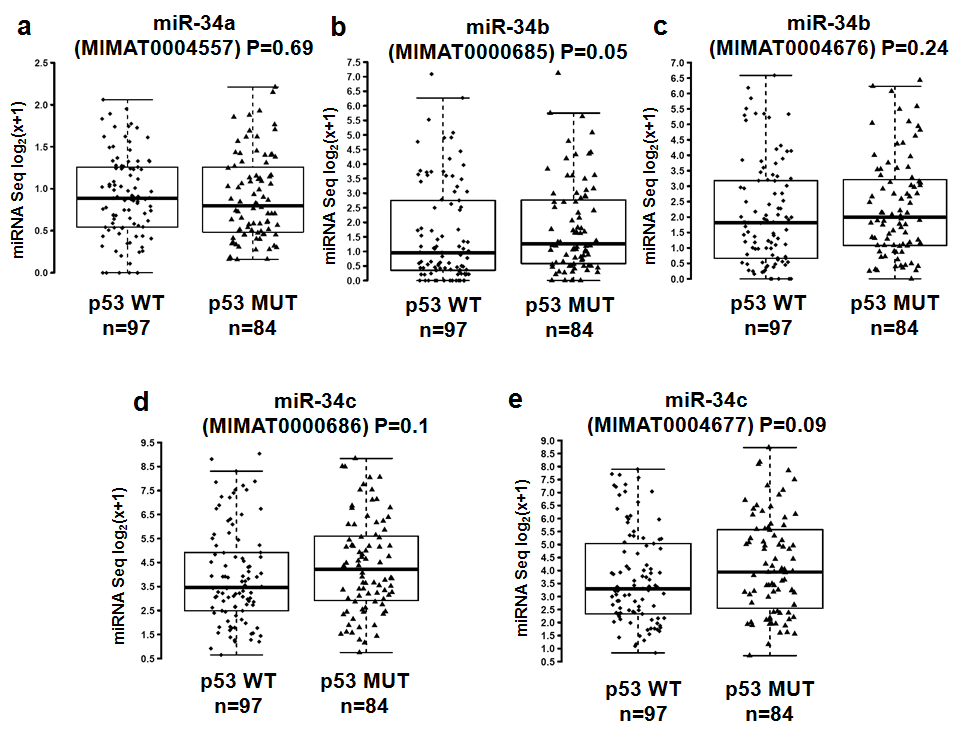


**Supplementary Figure 3**. miR-34a expression (a) and PDL1 (CD274) messenger RNA levels (b) in patients from the The Cancer Genome Atlas for Lung Adenocarcinoma (TCGA LUAD) based according to functional, partially functional , or non-functional p53 mutations. A box-and-whisker plot is used to represent the data. Box plot represents first (lower bound) quartile, median and third (upper bound) quartile. Whiskers represent 1.5 times the interquartile range. Kruskal–Wallis test. Two-sided. miRNA Seq= microRNA sequencing (TCGA); RNASeq Version 2= RNA sequencing version 2 (TCGA)

**
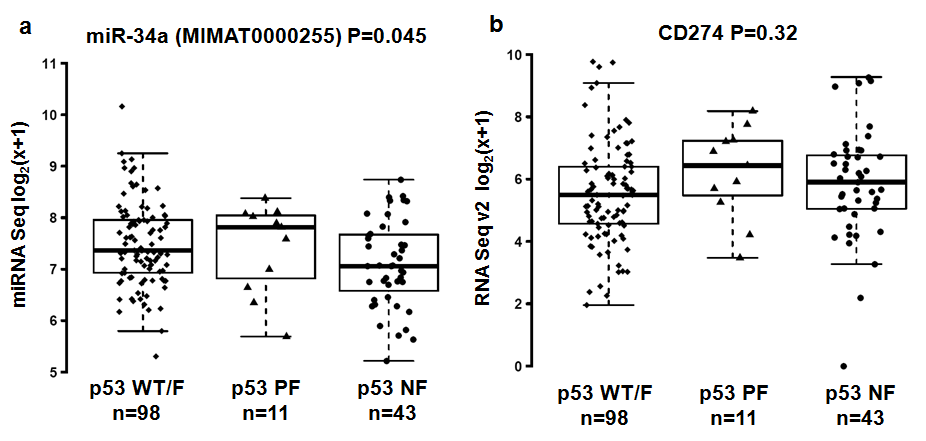
**

**Supplementary Figure 4.** Kaplan-Meier overall survival curves in the set of The Cancer Genome Atlas for Lung Adenocarcinoma (TCGA LUAD) patients according to miR-34a (a) and PDL1 (CD274) (b) expression. The number of patients at risk in low/high miR-34a (a) and PDL1 (CD274) (b) groups at different time points are presented at the bottom of the graph. Log-rank test, two-sided. OS=overall survival; (mo)=months; #=number.

**
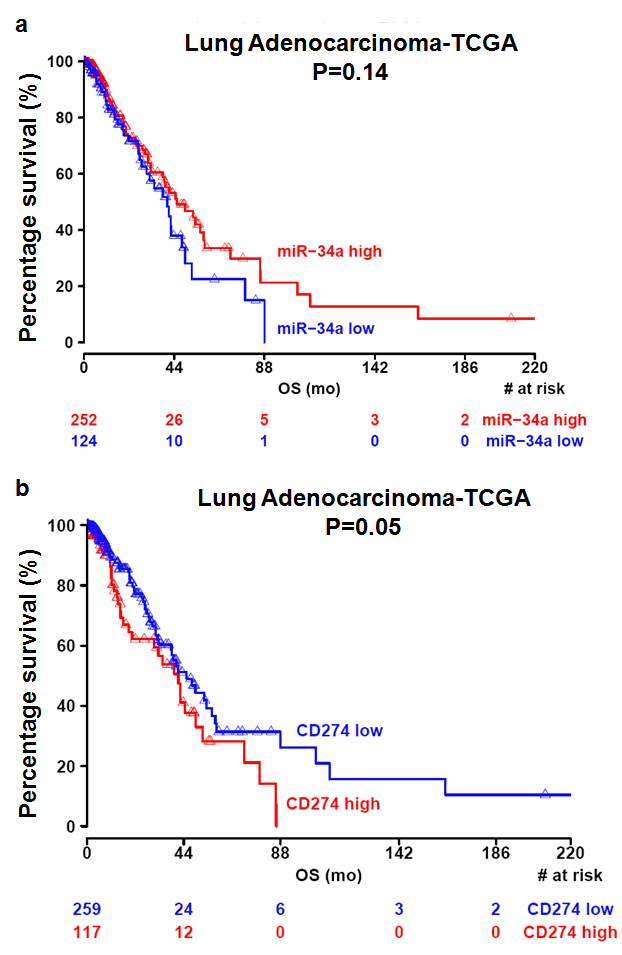
**

**
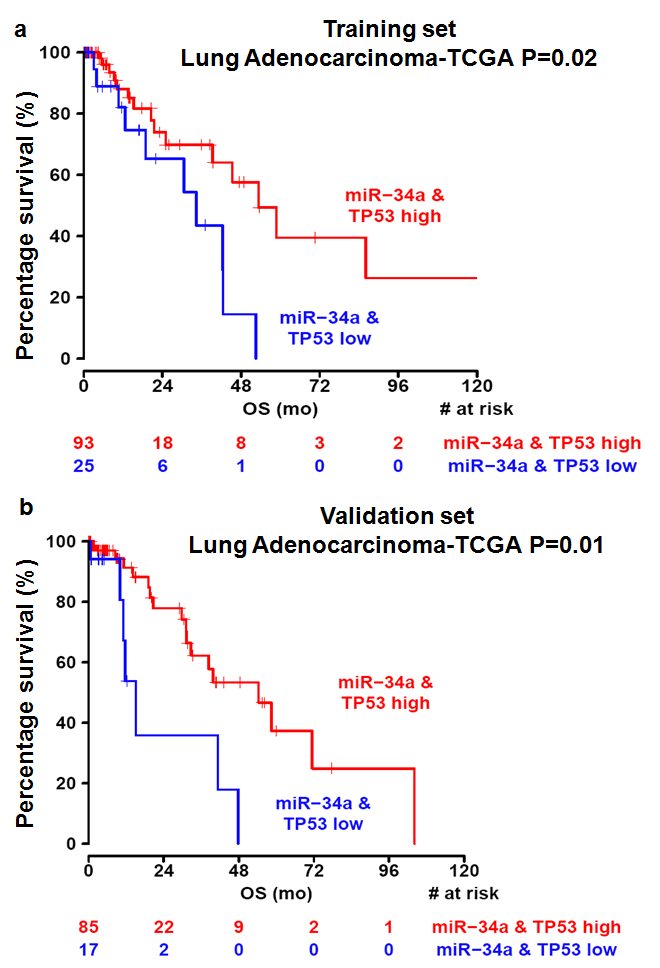
Supplementary Figure 5.**  Kaplan-Meier overall survival curves according to miR-34a and p53 expression in training (a) and validation (b) patients cohorts from The Cancer Genome Atlas for Lung Adenocarcinoma (TCGA LUAD). The number of patients at risk in low miR-34a/p53 and high miR-34a/p53 groups at different time points are presented at the bottom of the graph. Log-rank test, two-sided. OS=overall survival; (mo)=months; #=number.


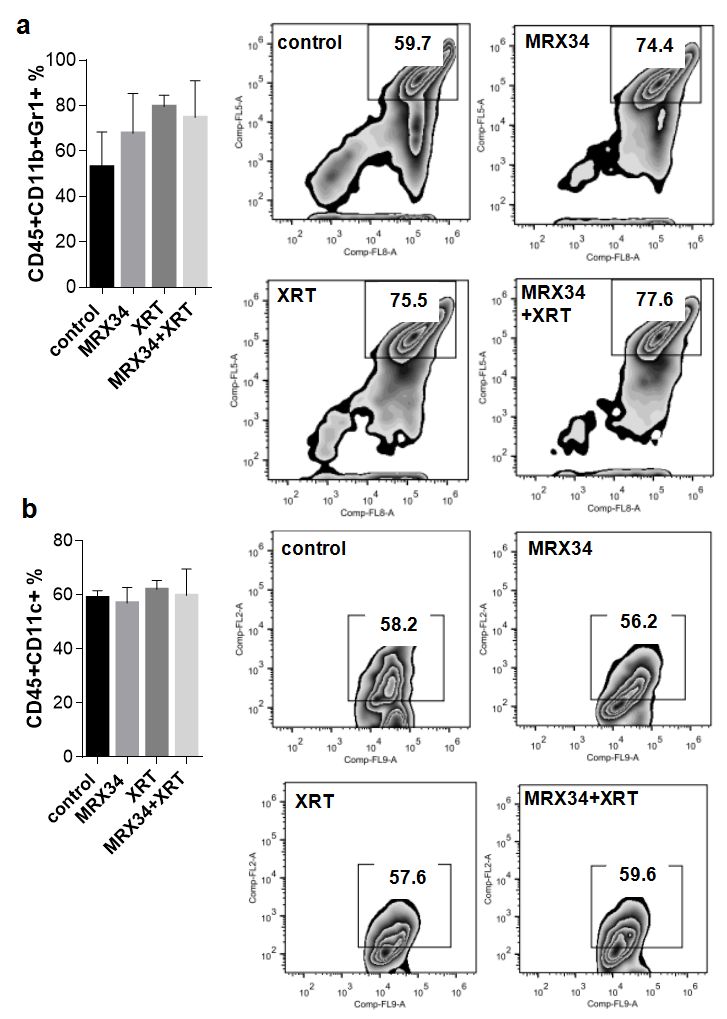
**Supplementary Figure 6.** Effect of miR-34a delivery in combination with radiotherapy (XRT) on dendritic cells (a) and myeloid-derived suppressor cells (b). Error bars on the bar charts represent standard deviation (SD). Comp-FL5-A=CD11b+; Comp-FL8-A=Gr1+
